# Relative age effects and player pathways in international ice hockey: a longitudinal multi-cohort analysis

**DOI:** 10.3389/fpsyg.2025.1583349

**Published:** 2025-07-29

**Authors:** Jean Lemoyne, Vladislav A. Bespomoshchnov, Mika Saarinen

**Affiliations:** ^1^Department of Human Kinetics, Université du Québec à Trois-Rivières, Trois-Rivières, QC, Canada; ^2^Department of Sport and Social Sciences, Norwegian School of Sport Sciences, Oslo, Norway; ^3^Haaga-Helia University of Applied Sciences, Vierumäki, Finland

**Keywords:** birth advantage, talent identification, long-term athlete development, competitive sport, high-performance, selection bias

## Abstract

**Introduction:**

Past research shows that relative age effects (RAEs) are highly prevalent in ice hockey. Early-born players benefit from more exposure, especially in the early stages of development, and are frequently considered “more talented.” Although RAEs are apparent in these early stages, little is known about how it affects pathways leading to the highest levels of competition. This study aims to look more closely at the associations between RAEs and players’ career trajectories in 4 hockey nations: Canada, Finland, Czechia, and Slovakia. Specifically, it aims to: (1) evaluate the prevalence of RAEs in each country, (2) identify players’ career pathways and examine the impact of RAEs on the players, and (3) compare these effects for each nation.

**Methods:**

Data were drawn from 4,306 players (100% males born between 1992 and 2002), who were invited to national development and selection camps between 2009 and 2019. Trajectory clusters were estimated from the players’ participation in 8 career milestones, from U17 to representation of their country at the Olympic Games. Group comparisons were conducted based on birth quartiles and hockey nations.

**Results:**

The results confirmed the presence of RAEs in the four hockey nations. Consistent with past research, early-born players are overrepresented in the early career stage, whereas late-born players begin to emerge during transition to junior level (U20). Some nation-specific differences were observed.

**Discussion:**

This provides further support for the stakeholders of ice hockey association looking to enhance their national team selection processes and discover structuring pathways that offer development opportunities for all groups of players.

## Introduction

Talent identification has been an important topic of interest in sport science for many decades. Professional sports organizations and national governing bodies across sports invest substantial financial and human resources in the search for players who can offer the team a competitive advantage. However, there is still a great degree of uncertainty when it comes to answering questions about the projected potential of any athlete (Baker et al., 2018). Plenty of examples across different sports point to players who are overlooked as junior athletes, then go on to become break-through success stories (Costello, 2022; Grella, 2023), which suggests that the timing of selections is crucial.

Despite the importance of such selections, many of these processes use pre-determined cut-off dates for player invitations to such events. Selected players can benefit from increased exposure including national/international and showcase-type tournaments. In a longer-term perspective, these athletes are described as more gifted in the earlier stages of development, which offers them opportunities to attain the next echelons of competition (Webdale et al., 2020). Among the factors influencing timing in selection processes are relative age effects (RAEs). RAEs describe the significant and often problematic consequences that arise from the common practice of grouping youth for participation and competition in sport based on chronological age, typically within one-year or two-year bands defined by arbitrary cut-off dates (Webdale et al., 2020). This organizational structure frequently leads to an over-representation of athletes born shortly after the cut-off date (i.e., the relatively older within their cohort) and an under-representation of those born later in the selection year (Webdale et al., 2020). This phenomenon has been documented across a wide array of sports (Bilgiç and Işın, 2023), starting from the seminal studies by Grondin et al. (1984) in ice hockey and volleyball and ice hockey specific conclusions from Barnsley et al. (1985). However, the expression and strength of RAEs are not uniform; they vary considerably across different sporting contexts and are influenced by a multitude of interacting factors including particular sport’s popularity, specific physical or cognitive demands of the sport, the competitive level, gender, and the particular age categories in question (Schorer et al., 2020).

Historically, RAEs have often been attributed primarily to the physical advantages, such as greater size and strength, that relatively older children may possess due to, on average, being more biologically mature than their chronologically younger peers within the same age group (Towlson et al., 2022). However, a substantial and growing body of evidence challenges this predominantly maturation-based explanation, suggesting instead that RAEs and biological maturity selection biases, while potentially related, are largely distinct and independent constructs (Towlson et al., 2022; Fitzgerald et al., 2024). Biological maturation selection bias refers to the preferential selection or deselection of individuals based on their advanced or delayed biological development, often irrespective of their birth date (Hill et al., 2020). This bias typically emerges with the onset of puberty, around 11–12 years of age, and its influence can increase as athletes progress through adolescence (Towlson et al., 2022; Fitzgerald et al., 2024; Hill et al., 2020). In contrast, RAEs are observable much earlier in an athlete’s development, often in pre-pubertal children as young as 6–8 years old, a period when maturational differences in physical attributes are less pronounced as selection factors (Fitzgerald et al., 2024; Hill et al., 2020). Recent research in sports like soccer and Gaelic football further underscores this distinction, revealing weak or non-significant correlations between an athlete’s relative age and their actual biological maturity status within academy settings (Towlson et al., 2022; Fitzgerald et al., 2024). This indicates that relatively older players are not necessarily the more biologically mature individuals in their cohort (Towlson et al., 2022; Hill et al., 2020).

This critical distinction necessitates a broader and more nuanced conceptualization of RAEs, viewing them as phenomena that extend beyond mere physical-maturational advantages (Towlson et al., 2022). In early childhood, for instance, RAEs may be more reflective of age-associated differences in neural development, cognitive skills, motor coordination, and the sheer volume of experience or practice time accumulated (Towlson et al., 2022; Hill et al., 2020). Furthermore, the initial, perhaps small, advantages afforded by relative age can be amplified by a cascade of secondary benefits on their developmental journey (Wattie et al., 2015). Athletes who are relatively older, and thus potentially perceived as more competent or “talented” early on, often receive greater expectancies, more positive feedback, increased attention from coaches, enhanced access to superior training resources, more significant playing opportunities, and higher quality coaching (Schorer et al., 2020; Hill et al., 2020; Lemoyne et al., 2023). This differential investment and experience can create a self-fulfilling prophecy, widening the gap between relatively older and younger athletes over time (Hancock et al., 2013). Conversely, relatively younger or later-maturing athletes, even if possessing high potential, face a greater risk of deselection and dropout, thereby missing crucial developmental opportunities (Towlson et al., 2022). Therefore, it is imperative to consider relative age and biological maturation as independent, though potentially interacting, influences within talent identification and development systems. Each of them likely requires distinct consideration and targeted mitigation strategies, implemented at appropriate developmental stages (Towlson et al., 2022; Fitzgerald et al., 2024).

As cited previously, Grondin et al. (1984) foundational work significantly advanced the understanding of RAEs. Subsequent research has confirmed and expanded on these findings. Lemoyne et al. (2023), for instance, revealed that RAEs are prevalent in most of the senior world’s top competitive ice hockey leagues. Furthermore, many authors also reported that RAEs were present in younger cohorts of competitive hockey players (Bruner et al., 2011; Niklasson et al., 2024; Lavoie et al., 2015; Lemoyne et al., 2021; Huard Pelletier and Lemoyne, 2022; Huard Pelletier and Lemoyne, 2024). It was suggested that the selection of players who appear more developed early on (often relatively older) is predicated on the motivation in finding individuals who are cable to perform on rather short-term basis (i.e., performance at the U16 or U17 tournaments) instead of focusing on athletes’ long-term projected potential (Niklasson et al., 2024; Sherar et al., 2007; Wattie et al., 2007). As a result, RAEs are likely to have an impact on gatekeeping in elite-level ice hockey like the National Hockey League and/or other levels of competition (Wattie et al., 2007; Fumarco et al., 2017; Deaner et al., 2013; Baker and Logan, 2007), although the accuracy of drafting-process decisions across sports has been questioned (Johnston et al., 2022).

In contrast, relatively younger players face some adversity, requiring them to adapt to the competitive system in which they develop as athletes. In this kind of developmental ecosystem, these players must compensate or *adapt to survive* (Baker and Johnston, 2024; Bruineberg et al., 2024). Two paradigms suggest how they adapt to the context shaped by RAEs. The first explanation, the psychological, stipulates that athletes born in the latter part of the selection year must develop stronger psychological assets (i.e., resilience, adaptability, grit) to overcome the disadvantages sometimes associated with their relative age. This hypothesis is supported in ice hockey by undrafted NHL players (Herbison et al., 2019), who are categorized as “sleepers,” whose talent tends to appear in the later stages of their career (Fortin-Guichard et al., 2023). The second explanation, the *biological-athletic*, suggests that to “survive” the selection biases associated with RAEs, relatively younger athletes may need to exhibit superior athletic skills predisposing them to adapt and excel in highly competitive contexts (McCarthy et al., 2016). Both explanations are plausible, and both may explain RAEs reversal in the later stages of the athlete’s pathway.

The reversal effects of RAEs stipulate that players born later in the year tend to equal or surpass those who were born earlier in the year at some point in their career trajectories. Despite the presence of RAEs at the junior level, many studies support the mechanisms underlying their reversal effects at the elite level (Lemoyne et al., 2023; Niklasson et al., 2024; Fumarco et al., 2017; Bezuglov et al., 2020). To date, however, it is not clear at what stage the reversal does begin to be such evident at the senior elite level (Steingröver et al., 2016). Schorer et al. (2020) highlights the need for studies to take a more longitudinal look at RAEs with recent examples from soccer (Brustio et al., 2024), handball (Schorer et al., 2025) and table tennis (Faber et al., 2020).

Hereby, this study takes a deeper look at the multi-cultural junior national team pathways leading to the highest levels of senior competition in ice hockey. The overarching aim of this article is to explore how RAEs relate to pathways leading players to the highest levels of competition in ice hockey. To achieve it, there are three objectives. The first objective is to evaluate the presence of RAEs in the selection processes of national teams in four countries: Canada, Czechia, Finland, and Slovakia. The second objective is to examine how RAEs relates to elite hockey players’ pathways to excellence from junior to senior national teams to estimate the phase where RAEs begin to reverse. The third objective of this study is to compare if RAEs and players’ pathways differ within and between hockey nations under study.

By examining the national team pathways of athletes in four nations, it refines our understanding of the pathways of athletes selected and/or de-selected across junior national team cohorts by evaluating their career trajectory clusters. Our hypothesis is based on the past and current literature on RAEs. We suggest that RAEs will be stronger in the early stages of career pathways, such as invitations to U17 and U18 national team camps. In line with Lemoyne et al. (2023), we suggest that the presence of RAEs will be similar across nations. Regarding the second objective, we expect to find some support for RAEs reversal at later stages of development (i.e., the professional level). We anticipate, consistent with this hypothesis, some transitional effects of RAEs related to the appearance of late-born players later in the process leading to national team selections.

## Methods

### Sample and data collection

Data were collected from male junior athletes who attended development or selection camps for junior national teams in four countries (Canada, Czechia, Finland and Slovakia), where ice hockey is a major winter team sport. Despite the popularity of ice hockey in these nations, there are still disparities in terms of registered players over 18 years of age [*n*_Canada_ = 132,169; *n*_Czechia_ = 6,622; *n*_Finland_ = 23,028; *n*_Slovakia_ = 3,332] (International Ice Hockey Federation, 2025, p. 63–64), which justifies the relevance of analyzing RAEs in such different development systems. The full sample for each nation includes players born between 1992 and 2002 who took part in national team development and selection camps, from invitation/selection of their national team for the World U17 Hockey Challenge (WU17), the World U18 Hockey Challenge (i.e., Hlinka Gretzky Cup), world junior championships (WJC), world hockey championships (WHC) and the Olympic Games (OG). Since playing in the National Hockey League is a key phase in hockey players’ careers, the players’ NHL draft status was considered a major indicator of their achievement. The professional career was tracked for 3 years following the last year of eligibility to perform at WJC (U20). This period was characterized by the NHL draft and performance in games at the international level (i.e., world championships and Olympic Games). We collected data from the websites of the national governing bodies of each nation and cross verified using open-source data from the *elite-prospects.com* website. When data was missing, we approached the ice hockey association of the respective nation to collect the missing data. The data collection and handling were conducted in strict compliance with general data protection guidelines. We safeguarded the identities of the participants included in this study and provided full confidentiality when reporting results.

### Variables

#### Birth quartile

We calculated birth quartile from the raw data available in each database. All birthdates were coded into birth quartiles (Q1 to Q4) based on the categories commonly used in ice hockey (International Ice Hockey Federation, 2025): (1) Q1: January to March; (2) Q2: April to June, (3); Q3: July to September; and (4) Q4: October to-December. As recommended by Delorme and Champely (2015), we checked to see if birth distributions were different in each country. Because of the prospective nature of our data, birth distributions were verified for each country for the period 1990–2002 by consulting the demographics and population statistics on each nation’s website. After verifying birth month distributions during this period, we observed no significant difference relative to birth month (proportions varying from 23 to 26% per quartile for each nation).

#### Quantifying hockey players’ career pathways

We defined the type of trajectory (or career pathway) by identifying players’ participation (or not) in eight stages of achievement in terms of national team selections and the NHL draft. As mentioned previously, the information was collected by extracting data from the websites and archives of each of the national governing bodies, with the addition of Open Access Internet Archive.^1^^,^^2^ First, we divided each stage of the hockey pathway based on player participation at each achievement milestone (*no* = 0; *yes* = 1). Second, we constructed a progressive trajectory starting with an invitation to a U16 national camp for the Europeans and a U17 national evaluation camp for the Canadians. To make all samples comparable, we chose U17 invitation camps as the first stage of players’ pathway (1st milestone) since there is no U16 invitation to national camps in Canada (no data available). This was followed by an invitation to U18 national camps (2nd milestone) and selection and participation in the U18 Hlinka Gretzky Cup (3rd milestone). The following stages included invitation to U20 national teams (4th milestone) and participation in the World Junior Championships (5th milestone). The three final milestones were recorded as the final stage of players’ trajectories. We chose the NHL draft status as 6th milestone because it usually occurs before national teams are invited to World Hockey Championships (7th milestone) and the Olympic Team (8th milestone) in the 3 years following the last year of U20 eligibility. The final database consists of 8 dichotomous variables (e.g., stages of achievement), which characterize the different types of career patterns.

Factor analyses were used to establish cut-off values for quantifying career pathways. Because of the exploratory nature of this study (and the resulting data), we chose Principal Axis Component as the analytical procedure. Given the (high) number of occurrences and potential variability for each career milestone, eigen values were fixed at 1.0 for dimension extraction. We also used varimax rotation to allow for factorial data structure; this is the appropriate procedure when anticipating uncorrelated indicators. Preliminary results were analyzed by evaluating the Kaiser-Meyer-Olkin (KMO) measure of adequacy, which was deemed satisfactory (KMO = 0.755). As a second step of our analysis, we determined the number of factor components by observing eigen values greater than 1.0. We also considered a scree plot to verify support for a three-component factorial structure.

The first factor (EV = 1.355) was defined as *early success* and includes three career milestones: U17 development camp (for all samples), invitation to U18 national camps, and participation in the U18 Hlinka Gretzky Cup. These loading components were strongly associated with their corresponding factor (λ_U17_ = 0.793; λ_U18inv_ = 0.773; λ_U18wc_ = 0.638). The second factor (EV = 2.949) was conceptualized as *junior transition* and includes three indicators considered career milestones: U20 invitation, U20 WJC, and NHL draft. Indicators around this dimension were correlated and displayed significant loadings (λ_U20inv_ = 0.810; λ_U20wc_ = 0.838; λ_NHL_ = 0.597). The third factor (EV = 0.950) was the *senior international pathway* and includes two career milestones: WHC participation and participation in the Olympic Games. Both indicators displayed strong loadings with their corresponding factor (λ_WC_ = 0.638; λ_OG_ = 0.630). After establishing this three-dimension factorial structure, factorial scores were calculated based on the regression method (Distefano et al., 2009). We then standardized factorial scores through z-transformation for each pathway. These scores attributed to each player reflect an overview of the dominant component of their career trajectories. They were then used for group comparisons and to verify the effects of RAEs on players’ pathways.

### Statistical analyses

To meet this study’s first objective (presence of RAEs), which consisted of evaluating the presence of RAEs across sub-samples, we conducted crosstabs analyses by calculating the chi-square (χ^2^) statistic for the full sample. We also compared RAEs prevalence in each nation to determine if RAEs proportions differ according to the hockey nations where players evolved. We used Cramer’s *V* to interpret the strength of association related to RAEs and interpreted values as weak (*V* = 0.10), moderate (*V* = 0.10–0.30) or large (*V* > 0.30) (Cramér, 1999).

As for the second objective of the study (RAEs and players’ pathways), we proceeded in two phases. First, we observed how the proportions of Q4 born players fluctuate at each milestone (e.g., from U17 to NHL). We excluded World Championships and Olympic Games numbers because there were too few observations, which would have potentially inflated the proportions for some countries. To proceed, we first applied Anscombe transformation on proportions and performed Analysis of Proportion using Arsine Transform (ANOPA). ANOPA, similar to classic analysis of variance (ANOVA), is designed for analyzing proportions as continuous variables (Laurencelle and Cousineau, 2023). To analyze the fluctuations of proportions, we tested monotonic variation which allows to see if variation is linearly significant. To base our interpretations, we used the coefficient of monotonicity (from 0 to 1) for the full sample (Laurencelle, 2021) and observed variation for each nation.

For the second phase of analysis (e.g., comparing pathways trajectories), we performed one-way ANOVA by comparing the factor scores. Due to potential violation of normality assumptions, we performed Bootstrapping (*n* = 1,000 iterations). We conducted *post hoc* analyses (with Bonferroni correction) in cases where differences were deemed significant. The magnitude of effect sizes was also interpreted according to eta-square values (*η*^2^), as small (*η*^2^ < 0.01), moderate (*η*^2^ = 0.06–0.14), and large (*η*^3^> 0.15) (Cohen and Cohen, 1983).

## Results

As Table 1 shows, an average of 391 players per year represented their team in international events. Canada (*M*_CAN_ = 101 ± 17) and Finland (*M*_FIN_ = 147 ± 39) tend to invite more players to national team camps, compared with Czechia and Slovakia who displayed lower numbers (*M*_CZE_ = 76 ± 9; *M*_SVK_ = 6 ± 7).

**Table 1:** Birth cohorts: the full sample (from available data).

Cohort (birth year)	Total (*n*)	Canada	Czechia	Finland	Slovakia
1992	447	104	81	193	69
1993	426	107	69	188	62
1994	469	105	74	214	76
1995	426	91	77	188	70
1996	385	117	78	134	56
1997	324	76	63	121	64
1998	329	83	70	117	59
1999	336	76	72	121	67
2000	397	116	98	119	64
2001	380	120	72	112	76
2002	387	123	82	117	65
Total	4,306	1,118	836	1,624	728

### Objective 1: examining presence of RAEs in the international context

**Figure 1:**
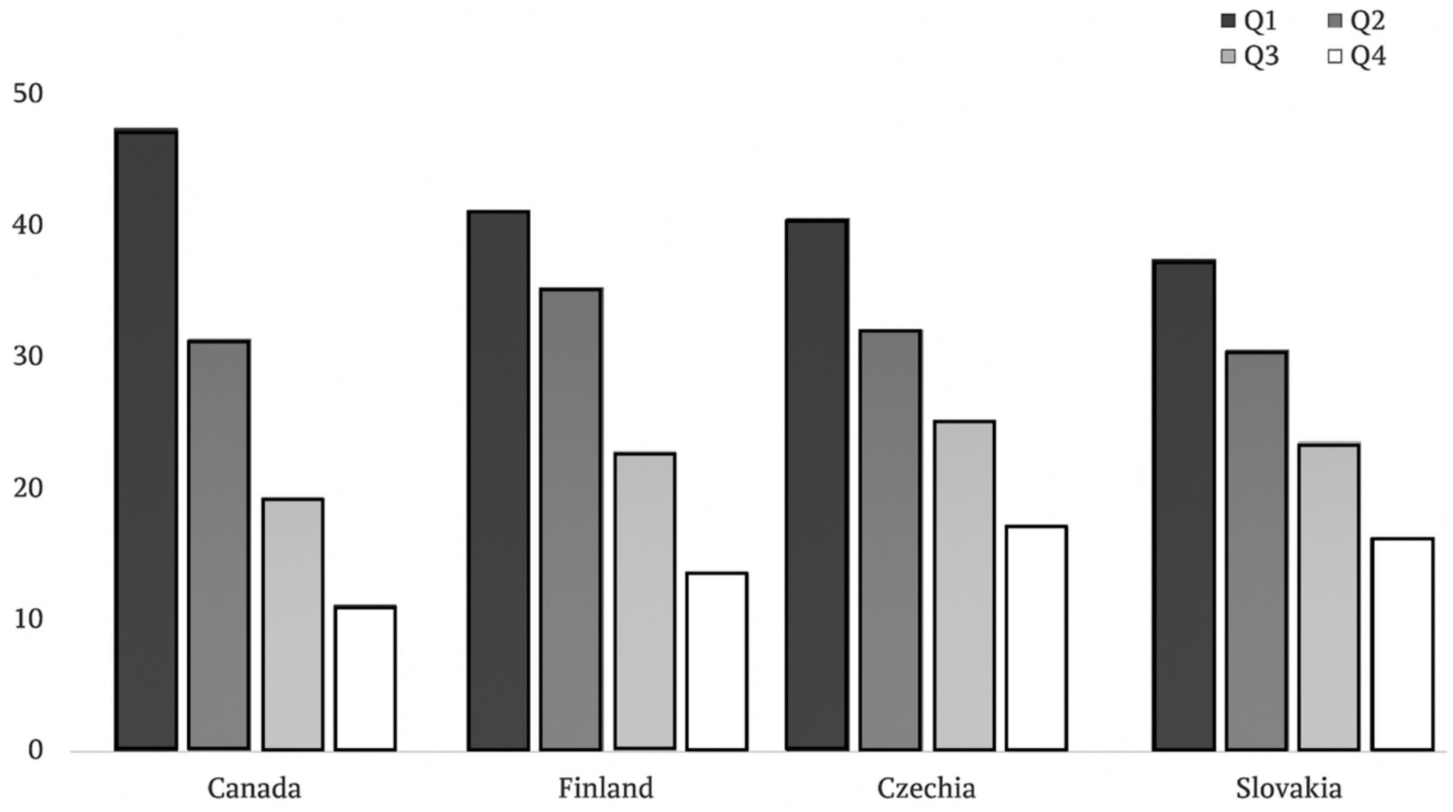
Birth quartiles patterns for each nation.

Figure 1 shows the proportions of players from each country in terms of birth quartiles. Generally, we observed similar tendencies in each sub-sample where Q1 (39%) and Q2 (29%) players were strongly represented, as compared with Q3 (20%) and Q4 (12%). In fact, RAEs are present across all samples [*χ*^2^_(df)_ = 719.16_(3)_, *p* < 0.001, Cramer’s *V* = 0.24] where Q1 and Q2 born players were predominant (68%). RAEs were observed in all sub-samples, with significant chi-square values and moderate to large effect sizes (*χ*^2^_CAN_ = 352.85, *p* < 0.001, Cramer’s *V* = 0.97; χ^2^_CZE_ = 86.58, *p* < 0.001, Cramer’s *V* = 0.55; χ^2^_FIN_ = 297.72, *p* < 0.001, Cramer’s *V* = 0.74; χ^2^_SVK_ = 45.67, *p* < 0.001, Cramer’s *V* = 0.43).

**Figure 2:**
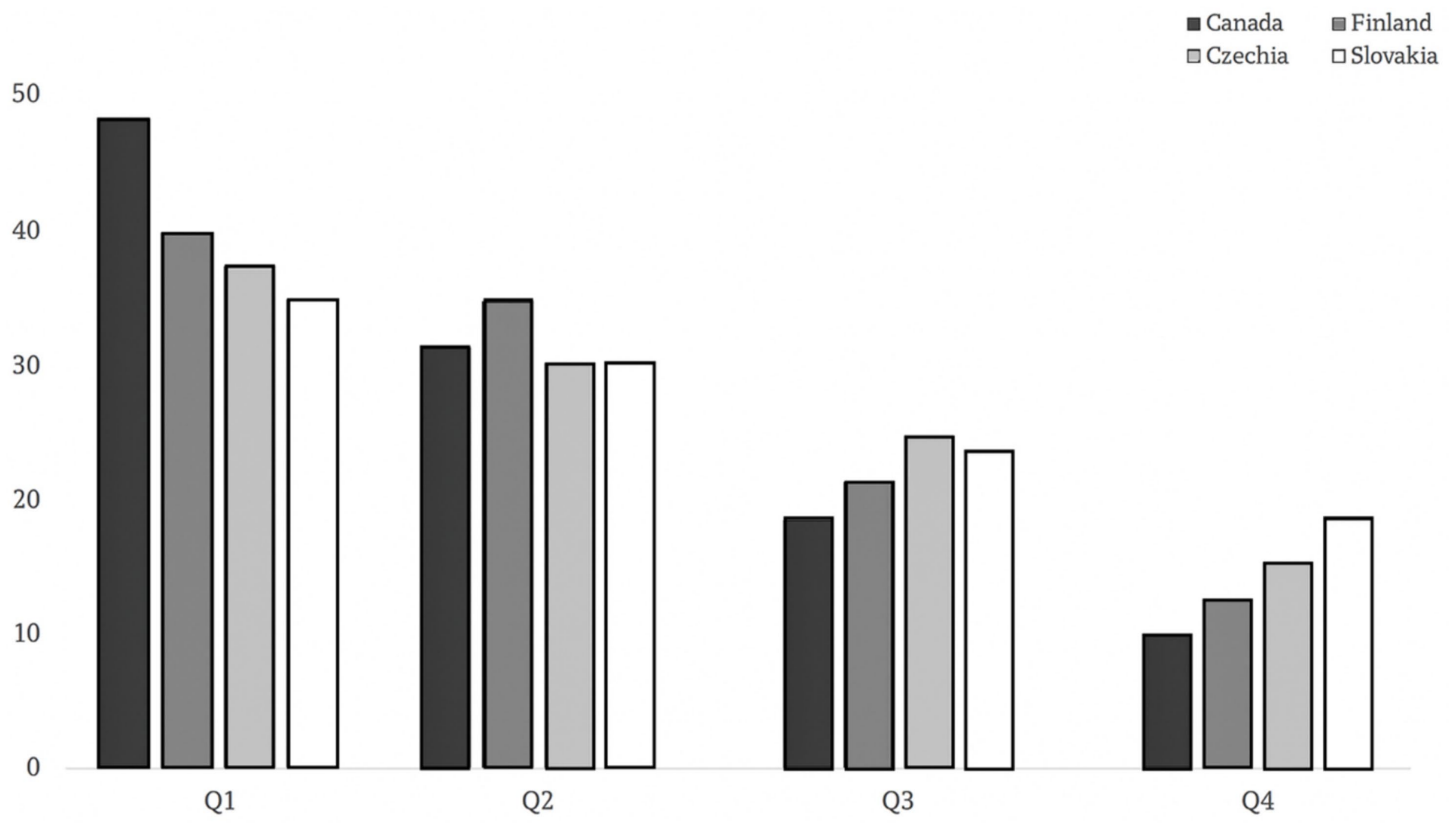
Proportions of players from each birth quartile according to nations.

However, some nation comparisons revealed significant differences [χ^2^_(df)_ = 70.73_(9)_, *p* < 0.001]. As Figure 2 illustrates, the Canadian sample displayed a higher proportion of Q1 born players (46%) compared to the European nations (between 32 and 38%). In addition, lower proportions of Q4 players were observed for Canada and Finland (9 and 11%), whereas the proportion of Q4 players was slightly higher across Slovakia and Czech samples (e.g., 14 and 18%). Cramer’s V was deemed small (*V* = 0.08, p < 0.001), suggesting small differences across countries.

### Objective 2: RAEs and players’ pathways

The second objective was to determine the impact of RAEs on players’ career trajectories. Table 2 provides a descriptive overview of the presence of late-born players (Q4) at each stage of career milestones. Results from monotonic variation test suggest a linear increase (from U17 to the attainment of the NHL): Monotonic variation coefficient = 0.93; *p* < 0.01. When we compared nations separately, Canada and Czech fluctuations were not monotonic (Spearman *r*_CAN_ = 0.83; Spearman *r*_CZE_ = 0.54, *p* > 0.10), whereas those of Finland and Slovakia increases were linearly significant (Spearman *r*_FIN_ = 0.95; Spearman *r*_CZE_ = 1.00, *p* < 0.001).

**Table 2:** Proportions of late-born players (Q4) across each career milestone.

	Career milestones							
Nation	U17	U18_inv_	U18_HC_	U20_inv_	WJC	NHL	WC	OG
Canada	69 (7%)	27 (6%)	11 (5%)	37 (15%)	21 (11%)	50 (11%)	18 (17%)	5 (41%)
Czechia	71 (14%)	65 (14%)	29 (17%)	58 (18%)	30 (19%)	10 (14%)	3 (17%)	0 (0%)
Finland	56 (10%)	47 (13%)	21 (13%)	48 (14%)	26 (15%)	21 (15%)	10 (17%)	1 (6%)
Slovakia	61 (14%)	74 (19%)	37 (19%)	61 (21%)	37 (22%)	6 (22%)	11 (34%)	3 (42%)

Values represent proportions of players who achieved each career milestone in numbers (n) and (%). Numbers represent proportions of late-born players who took part in the corresponding milestone in the years following their eligibility to U17 national camps. Milestones descriptions: U17: U17 national camp; U18_inv_: invitation to national U18 camp; U18_HC_: participation in Hlinka Gretzky Cup; U20_inv_: invitation to national junior team camp; WJC: World junior championships; NHL: drafted in National Hockey League; WC: World senior championships; OG: Olympic Games.

**Figure 3:**
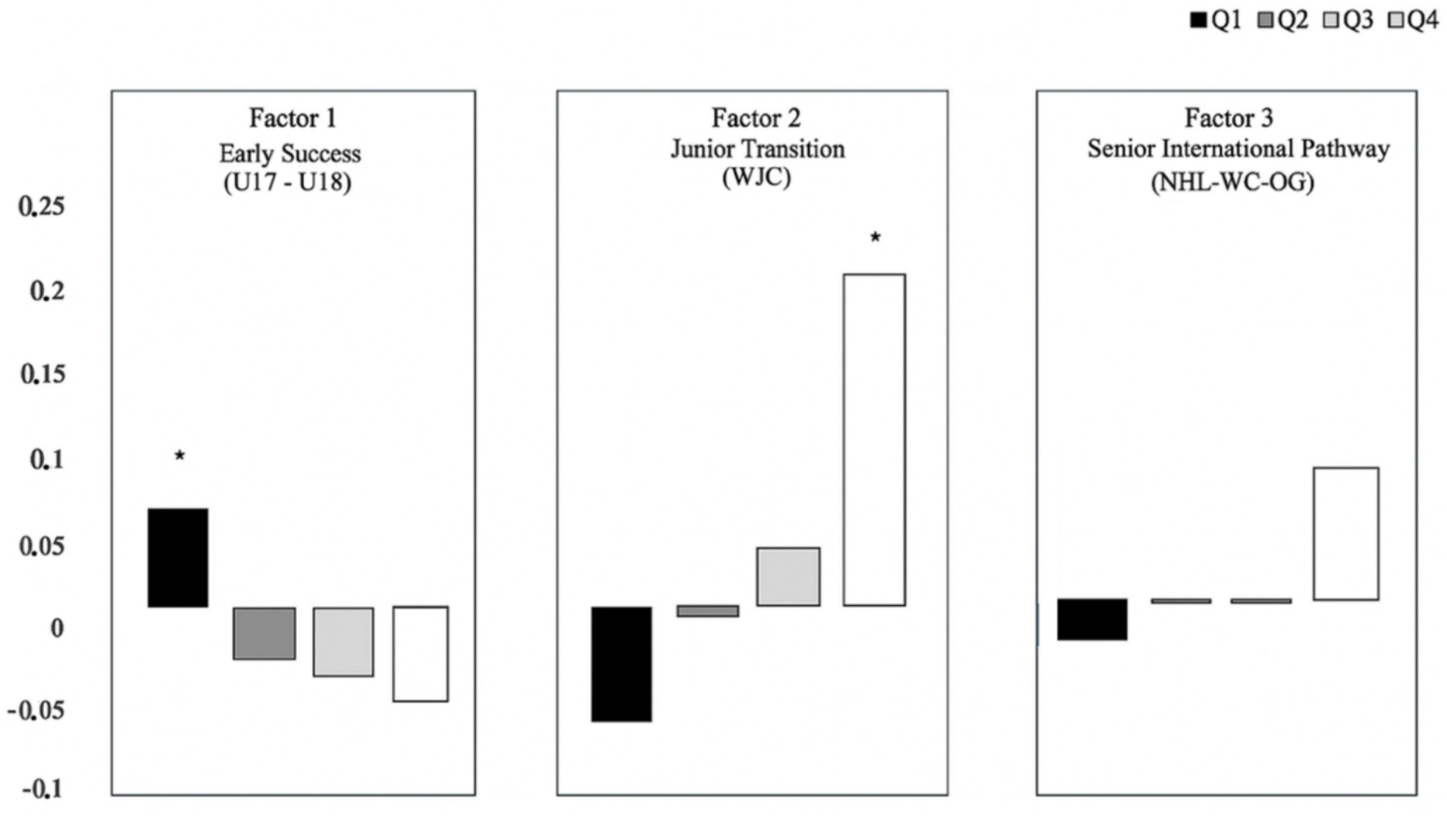
Birth quartile and early career trajectories (normalized factor scores according to birth quartile).

As Figure 3 depicts the career trajectory patterns, Table 3 shows the associations between RAEs and factorial scores for the full sample (e.g., including all nations). Results reveal a significant RAEs for two factors: Factor 1 (*early success: F*_early_ = 3.73, *p* < 0.011). and Factor 2 (*junior transition: F*_junior_ = 11.21, *p* < 0.001).

**Table 3:** RAEs and career achievement in elite hockey (standardized scores).

Career trajectories	Q1	Q2	Q3	Q4	Effects
F1	0.063 ± 0.98	−0.033 ± 1.01	−0.042 ± 1.01	−0.060 ± 1.02	Q1 > Q4*
F2	−0.075 ± 0.96	−0.005 ± 0.96	0.032 ± 1.04	0.210 ± 1.11	Q4 > Q1*
F3	−0.022 ± 0.86	−0.002 ± 0.91	0.003 ± 1.13	0.084 ± 1.39	No differences^ns^

F1: early success; F2: junior transition; F3: senior international pathway. *p < 0.001: significant differences between quartiles.

In line with RAEs hypotheses, comparisons regarding Factor 1 (e.g., *early success*) show that early-born players (e.g., Q1) have higher scores than Q3 and Q4 players: *Δ* > 0.04–0.22, *p* < 0.05. These differences were also interpreted as small (η^2^_early_ = 0.003). For Factor 2, *post hoc* analyses of birth quartiles show that late-born players (Q4) display higher scores (e.g., *junior transition*) than players born in the previous birth quartiles: Δ > 0.17–0.36, *p* < 0.001. Despite its significance, we interpreted the magnitude of this difference as small (η^2^_junior_ = 0.008). We found no significant differences for Factor 3 (e.g., *senior international pathway*): *F*_int_ = 1.51, *p* = 0.21, η^2^ = 0.001.

### Objective 3: nation comparisons: RAEs and career pathways

ANOVA results suggest that some nation-related differences prevail concerning RAEs on players’ pathways (see Table 3). Analyses regarding Factor 1 (e.g., *early success*) show support for RAEs in the Canadian sample: *F*_early-Can_ = 9.17, *p* < 0.001, *η*^2^ = 0.024. However, no such effects were observed in the European countries: *F*_early-Cze_ = 0.36; *F*_early-Fin_ = 0.27; *F*_early-Svk_ = 0.88, all at *p* > 0.45. This means that despite the greater representation of Q1 players in terms of proportions, there is no RAEs on the *early success* component of European players’ pathways. For Factor 2 *(junior transition),* we observed significant effects for three countries: *F*_Can_ = 3.35, *p* = 0.018, *F*_Fin_ = 4.19, *p* = 0.006; *F*_Svk_ = 3.90, *p* = 0.009. *Post hoc* analyses showed that Q4 players tend to surpass Q1 players at this specific stage of development. However, there were no RAEs in Czechia’s sample: *F*_Cze_ = 1.17, *p* = 0.17. For Factor 3 (senior international pathways, we found no significant differences in regard with birth quartiles: *F* = 0.1.51, *p* = 0.21; *η*^2^ = 0.001. When we compared each nation separately, Q4 players displayed higher scores than the three other birth quartiles: *F* = 8.51, *p* < 0.001, *η*^2^ = 0.022 (Table 4).

**Table 4:** RAEs and career achievement in elite hockey by country.

Country		Q1	Q2	Q3	Q4	Effects
Canada (*n* = 1,118)	F1	0.324 ± 0.04	0.175 ± 0.05	0.100 ± 0.06	−0.144 ± 0.09	Q1 > Q3*, Q4**
Canada (*n* = 1,118)	F2	−0.027 ± 0.06	0.083 ± 0.07	0.222 ± 0.09	0.328 ± 0.127	Q4 > Q1*
	F3	0.183 ± 0.05	0.298 ± 0.06	0.167 ± 0.08	0.791 ± 0.11	Q4 > Q1, Q2, Q3**
Czechia (*n* = 836)	F1	0.312 ± 0.06	0.228 ± 0.06	0.292 ± 0.07	0.244 ± 0.09	No effects^ns^
Czechia (*n* = 836)	F2	−0.011 ± 0.06	0.049 ± 0.06	0.019 ± 0.07	0.221 ± 0.07	No effects^ns^
	F3	−0.193 ± 0.03	−0.200 ± 0.03	−0.149 ± 0.04	−0.247 ± 0.05	No effects^ns^
Finland (*n* = 1,624)	F1	−0.385 ± 0.04	−0.400 ± 0.04	−0.441 ± 0.05	−0.397 ± 0.07	No effects^ns^
Finland (*n* = 1,624)	F2	−0.153 ± 0.32	−0.132 ± 0.35	−0.076 ± 0.45	0.082 ± 0.06	Q4 > Q1**
	F3	−0.079 ± 0.04	−0.017 ± 0.05	0.035 ± 0.06	−0.040 ± 0.08	No effects^ns^
Slovakian (*n* = 723)	F1	0.328 ± 0.07	0.304 ± 0.07	0.205 ± 0.09	0.172 ± 0.09	No effects^ns^
Slovakia (*n* = 723)	F2	−0.066 ± 0.06	0.116 ± 0.07	0.038 ± 0.08	0.284 ± 0.09	Q4 > Q1**
	F3	−0.102 ± 0.07	−0.210 ± 0.08	−0.119 ± 0.09	−0.023 ± 0.09	No effects^ns^

*p < 0.05; **p < 0.01. F1: early success; F2: junior transition; F3: senior international pathway.

## Discussion

This study first aimed to evaluate whether RAEs are present among a cohort of junior players in Canadian, Finnish, Slovakian and Czech national teams. Our second objective was to examine how RAEs relate to elite hockey players’ pathways to excellence from junior to senior national teams and estimate the phase where RAEs begin to reverse. The third objective of this study is to compare if RAEs and players’ pathways differ within and between hockey nations under study. To this end, we quantified players’ career trajectories by taking a deeper look at the different stages leading to professional and international careers and which involve three specific milestones: early success, transition to junior level and senior international pathway. By considering multiple career milestones at once, this analytical approach allowed us to verify the impacts of birth month on career achievement during early career stages, resulting in three specific observations that help shed light on talent selection (and development) in team sports. They include: the persistence of RAEs on the international stage, the impact of RAEs on career trajectories, and the presence of country-specific differences.

### Observation 1: RAEs are (still) present on the international stage

As initially anticipated, results indicated that RAEs are present in all four hockey countries in our investigation, and are congruent with those of past research, suggesting that RAEs are highly prevalent in (popular) team sports like soccer and ice hockey (Lemoyne et al., 2023; Brustio et al., 2024). This prevalence is observed for the full sample, which confirms that early-born players (e.g., Q1 and Q2), representing nearly 70% of this sample, are predominant. Given that the data derive from an 8-year follow-up period of 11 birth year cohorts (2002–1992), this suggests that RAEs persist through time, a recognized fact for the last 40 years (Grondin et al., 1984; Barnsley et al., 1985). A few explanations support our results. First, we must consider the context in which the samples were selected. Considering the high number of players invited to U17 development camps (and U16 in European countries), we believe early born advantages may be inflated because of the need to perform (or stand out) early. Considering evidence from previous research in ice hockey (Lemoyne et al., 2023; Lemoyne et al., 2021), larger numbers of these types of players were expected. In fact, the byproducts of RAEs such as the complexity of interaction between physical, neural, psychological and cognitive development, play a major role in sports like hockey, and those with these developmental assets at an early stage of development is likely to possess a significant advantage over others (Lemoyne et al., 2021). Since the RAEs appear to be an enduring phenomenon in ice hockey, we consider these dynamics natural to some extent. The results of this investigation show that coaching staff and association’s stakeholders tend, understandably, to prioritize assembling the strongest possible teams for international competitions, especially during the early stages of international tournaments (U17 and Hlinka Gretzky Cup). These actions then reinforce the presence of RAEs in international ice hockey despite many publications warning about this bias in player selection across sports (Cohen and Cohen, 1983) and particularly ice hockey. Hence, it could be argued that national ice hockey associations need to consider re-orienting the paradigm of player selection and development (Güllich and Barth, 2024).

### Observation 2: RAEs impact career trajectories

The three trajectories identified in our analysis were affected by RAEs at different levels. The first factor (*early success*) included milestones regarding selection to U17 development camp (for all samples), invitation to U18 national camps, and playing in the U18 Hlinka Gretzky Cup. The second factor (*junior transition)* included three indicators we view as career milestones: U20 invitation, U20 WJC, and NHL draft. The third factor (*senior international pathway*) included two career milestones: WHC participation and participation in the Olympic Games. Our results are consistent with the hypothesis of RAEs reversal at later stages of development. This is supported by two key stages of players’ pathways to the highest levels of competition. Accordingly, this study shows that successful transition to junior level may be the first stage when RAEs reversal (or fading) occurs. Late-born (Q4) players displayed higher scores in this crucial phase of development (U20 and NHL draft), so it appears they survived the RAEs. This RAEs reversal may be explained in part by late-born players’ ability to adapt to and survive in a highly competitive environment (Fumarco et al., 2017; Steingröver et al., 2016). Inversely, a substantial proportion of early-born players (Q1, Q2) tend to lose these physical advantages, making it difficult for them to adapt to the next level. Results indicate that Q4 born players begin to emerge during the successful transition to junior level, which coincides with a time when the physical advantage may be less obvious, and other qualities such as skilled engagement with the puck, dexterity (movement problem solving; Bernstein, 1996), and psychological or perceptual-cognitive factors may come to the fore. Results for Factor 3 show no differences and offer additional support for RAEs reversal at the international stage (McCarthy et al., 2016). Indeed, no significant differences were observed for the birth quartiles. This means that even at early career stages (8-year timeline - from U17 to early participation in world class events), no advantages derive from birthdate. This suggests a reversal has already occurred earlier in the international career, which is likely the introduction to international junior hockey. Finally, our results support the presence of a clear advantage for early-borns (Q1) in terms of RAEs in the early stages of elite players’ pathways (U17, U18), where Q1 players are over-represented. Labelled *early success*, the findings align with past research revealing that players born in Q1 have numerous advantages (neurological, cognitive, biological and psychological) over those born later in the year and may be the national team’s preferred choice in the short term due to their physical maturity.

Observation of RAEs reversal during the years of junior transition leads to a call to action. First, we maintain that, rather than structure international pathways around the quest to win, decisionmakers should emphasize the creation/implementation of comprehensive player development programs (Wattie et al., 2015; Till and Baker, 2020). As it is crucial to acknowledge that winning alone is not necessarily a sign of success and knowing that reversal seems to occur during the key stage of junior transition, we should continue to think about finding ways to discover “latent” talent (Fortin-Guichard et al., 2023). In fact, involving larger number of players annually and increasing players’ turnover by exposing them to high-level competitive environments may be a practice worth promoting. Based on our results, federation-led initiatives should be extended and reinforced at U17, U19, and even U21 level events. For example, international game breaks present an excellent opportunity to organize “shadow tournaments” or showcases for players not currently representing their country. In other contexts, special camps for these kinds of players (e.g., late-maturing, late-born) could be organized to allow them to overcome previous disadvantages. These approaches would offer both players and governing bodies the opportunity to discover emerging talents, making the development pathway deeper and potentially more efficient.

### Observation 3: international differences in RAEs patterns

Considering the unique constraints of each country in this study, it’s important to consider the impact they have on the factors discussed here (Røsten et al., 2024). The predominance of Q1 players in the early stages of national team camps (in terms of proportion) was similar for each nation. Despite these similarities, we observed some different patterns regarding the effects of birth month on players’ pathways. As for the *junior transition* factor, we note that late-borns displayed higher scores, which seems to correspond to the stage when they could overcome their previous disadvantage. Results indicate that this effect was stronger among the Finnish and Slovakian cohorts, whereas it was a tendency for Canadian players. A possible explanation is that the player pool, throughout the career pathway, provides more players invited to national events during the later stages of the junior national team pathway. A look at the Finnish sample size shows that despite the smaller total size of registered players, Finns tend to invite more players (relative to their number of available players) at this specific stage of development. No effects of RAEs were seen in the Czech sample, which could be due to a smaller pool of available elite players. Inversely, the RAEs on players’ pathways was deemed significant at all stages in the Canadian sample. The strong RAEs on early success came as no surprise, considering the number of highly competitive players across the country. Additionally, the sociodemographic aspects of the organization of ice hockey in Canada are also to be considered explanations. Since Canada is a vast territory, it has multiple provincial branches that work in different contexts. Nevertheless, it was interesting to note the gradual disappearance of RAEs during the elite junior years, which is similar to international junior hockey and the NHL draft. These results must be taken into account in future to persuade stakeholders in highly competitive hockey that this is a crucial stage for the potential emergence of late born-players (Niklasson et al., 2024; Wattie et al., 2007).

### Future perspectives

This study’s findings underscore that the predominant trend in international junior ice hockey involves selecting early-born players at first national team selection events at the age of 15 or 16 (depending on the nation). Following the COVID-19 pandemic, the Finnish Ice Hockey Association decided to delay the time of first selection to the first national team (U16) by 7 months. The long-term impact of the decision has yet to be studied. For practical reasons, we believe an initiative of this kind should be encouraged as the foundational problem in the talent pathways is the practice of early selection itself. This premature filtering of athletes creates a systemic vulnerability to bias, where factors like RAEs and differences in biological maturation become primary determinants of success. These factors should not be viewed as the cause of the problem, but rather as prominent symptoms of a system fundamentally flawed by its focus on selection. When performance at a single moment is the primary criterion, the system inevitably favors athletes who are temporarily advantaged, leading to significant survivorship bias. Consequently, interventions such to mitigate these biases, while promising, are inherently limited; they attempt to manage the symptoms without confronting the underlying issue, which is the philosophical commitment to identifying talent through early selection.

Some nations have recently begun to integrate a U19 Challenger team to enlarge the pool of invited players to U20 training camps. Hence, a similar strategy could be adopted at earlier stages of development (U16, U17, U18), allowing young athletes to improve the different skills related to sport development. Every nation has its own constraints in terms of organizational constraints, yet more players in the national team pipeline would allow ice hockey governing bodies to expose greater number of athletes to better-quality coaching and training opportunities as well as a greater number of competitive games at the international level (Till and Baker, 2020).

### Limitations and research recommendations

This study only covers the 3 years since the last year of eligibility to play in U20 international tournaments. Hence, future studies need to consider a more longitudinal trajectory and study athlete’s pathways from the beginning to end of their playing career to obtain a more complete picture. We recognize, furthermore, that player development occurs mainly at the club level, but we only considered athletes’ national team pathway. In the future, studies should take into consideration club or team level. It would allow us to acquire a more nuanced understanding of athletes’ development years. This suggestion may apply more to the European cohorts owing to the structure of the club and competitions. Future research should also take into consideration players’ performance and integrate relevant metrics to capture the potential gap between international and club level performance. Finally, while we looked at the outcomes of the selection decisions of junior national team coaches and scouts, we did not consider socio-cultural aspects such as the playing style preferences of each country and/or the national values that might impact decisions about the players (Røsten et al., 2024).

## Conclusion

Although ice hockey shows overwhelming evidence of RAEs at the junior level and its reversal effects at the elite level (Lemoyne et al., 2023), the policy of sports governing bodies to “fight” this bias has changed minimally, despite some attempts to delay selection time. Therefore, we propose that a fundamental philosophical shift is required, moving away from a focus on early talent identification and toward a holistic, development-centered model. Adopting a different approach that recognizes individual and non-linear developmental pathways. The primary purpose of junior sport should be redefined as maximizing long-term development for all participants, not selecting the best performers early. By postponing high-stakes selection until athletes are older would diminish the impact of selection biases associated with RAEs and maturation. This paradigm shift necessitates a complete re-evaluation of youth sporting structures, from coaching pedagogy to the nature of competition, to create an environment that prioritizes learning and growth over immediate results, ultimately ensuring a more equitable and effective pathway for talent development. Thus, we hope this work will help associations and professional sports organizations reflect on the possible competitive advantage to be gained from delaying the selection of athletes and broadening the pool of players to provide opportunities for the athletes born later in the year to flourish instead of dismissing them early on the pathway.

## Data Availability

The original contributions presented in the study are publicly available. This data can be found here: https://osf.io/u6xqr/?view_only=23e0e27faee74598833a5faba325b0cc.

## Footnotes

^1^ https://eliteprosects.com

^2^Hockey Canada website.
